# Association of uric acid, HDL-C, and homocysteine with coronary artery disease and stenosis

**DOI:** 10.3389/fcvm.2026.1765538

**Published:** 2026-07-20

**Authors:** Lei Zha, Yu Ma, Lianghui Wan, Pingan Xie, Ximing Li

**Affiliations:** 1 Cardiology Department, Tianjin Chest Hospital, Tianjin, China; 2 Cardiology Department, The First Affiliated Hospital of Tianjin University of Traditional Chinese Medicine, Tianjin, China

**Keywords:** coronary artery stenosis, coronary heart disease, high-density lipoprotein cholesterol, homocysteine, uric acid

## Abstract

**Background:**

Coronary heart disease (CHD) remains a major global cause of morbidity and mortality. Identifying reliable and non-invasive biochemical markers that reflect CHD severity may improve early diagnosis and risk stratification. This study investigated the associations of homocysteine (Hcy), high-density lipoprotein cholesterol (HDL-C), and uric acid (UA) with CHD severity, and evaluated the diagnostic value of their combined detection.

**Methods:**

A total of 100 participants undergoing coronary angiography were enrolled, including 58 patients with CHD and 42 controls. Serum Hcy, HDL-C, and UA were measured, and coronary stenosis burden was quantified using the Gensini score. Spearman correlation and multivariable linear regression analyses were performed to identify independent predictors of Gensini score. Receiver operating characteristic (ROC) curves and reclassification indices—including net reclassification improvement (NRI), integrated discrimination improvement (IDI), and *Δ*AUC—were used to assess diagnostic performance. Best subset regression evaluated biomarker selection.

**Results:**

Hcy, UA, and HDL-C were significantly altered in CHD patients compared with controls (all *p* < 0.001). Gensini scores were positively correlated with Hcy (r = 0.314), UA (r = 0.307), Scr (r = 0.411), NT-proBNP (r = 0.294), and fasting glucose (r = 0.252), but negatively correlated with HDL-C (r = –0.324; all *p* < 0.01). In multivariable models, adding Hcy, UA, and HDL-C to clinical predictors increased the explained variance of the Gensini score from 31% to 48% (Model 2 R^2^ = 0.48, *p* < 0.001). Individually, Hcy showed the highest diagnostic value (AUC = 0.794), followed by HDL-C (AUC = 0.767) and UA (AUC = 0.749). The combined biomarker model achieved the highest discriminative performance (AUC = 0.803; sensitivity 69.8%, specificity 74.2%). Reclassification metrics demonstrated modest improvement with the combined model (NRI = 0.24, IDI = 0.065, *Δ*AUC = 0.084; all *p* < 0.01). Best subset regression and BIC criteria consistently supported Hcy, UA, and HDL-C as optimal predictors.

**Conclusion:**

Serum Hcy, UA, and HDL-C are significantly associated with both the presence and severity of CHD. Their combined detection provides a modest improvement in diagnostic accuracy and improves risk stratification beyond traditional clinical variables. These biomarkers may aid early assessment of coronary stenosis burden.

## 1 Introduction

Coronary heart disease (CHD) and coronary artery stenosis (CAS) present major global public health challenges, impacting over 400 million individuals worldwide (1). According to the World Health Organization (WHO), CHD remains one of the leading causes of death among adults, with its incidence and mortality rates rising due to socio-economic development and an aging population (2, 3). The pathogenesis of CHD is multifaceted, primarily involving CAS, which results in inadequate myocardial blood supply, hypoxia, and potentially myocardial infarction. The risk of CHD is influenced by various factors, including poor dietary habits, chronic stress, smoking, physical inactivity, overweight and obesity, as well as metabolic disorders, inflammatory responses, dyslipidemia and oxidative stress (4–6).

Coronary angiography (CAG) is the “gold standard” for diagnosing CHD, but this examination is highly invasive and has high risks and costs (7). Recent advances in non-invasive imaging techniques, including CTA, CMR, and echocardiography, have improved diagnostic accuracy but remain limited by equipment cost and operator expertise, especially in resource-constrained settings (8, 9). Consequently, there is growing interest in developing simple, low-risk, and accessible diagnostic strategies. In clinical practice, alternative non-invasive diagnostic approaches, particularly blood biochemical tests, are increasingly recognized for their ability to provide straightforward assessments of a patient's cardiovascular health (10, 11). Although previous studies have investigated individual biomarkers, few have systematically evaluated the combined predictive value of homocysteine (Hcy), uric acid (UA), and high-density lipoprotein cholesterol (HDL-C), particularly in relation to coronary stenosis burden and patient stratification by clinical risk factors such as age, gender, smoking, and statin use. This represents a novel aspect of our study, as integrating these biomarkers with contemporary statistical approaches, including multivariable regression, receiver operating characteristic analysis, and reclassification metrics (NRI, IDI, *Δ*AUC), allows for a more precise non-invasive prediction of CHD severity. The purpose of this research is to investigate the correlations between high-density lipoprotein cholesterol (HDL-C), homocysteine (Hcy), and uric acid (UA) in individuals with CHD severity. We hypothesized that: (1) patients with CHD would exhibit significantly altered levels of these three biomarkers compared to those without CHD; (2) the levels of these biomarkers would correlate with coronary stenosis burden as measured by the Gensini score; and (3) the combined assessment of these three biomarkers would provide a more accurate non-invasive diagnostic approach for CHD severity compared to individual biomarker evaluation. By systematically analyzing combined biomarker profiles and applying updated statistical methodologies, this study offers a contemporary and clinically relevant approach to non-invasive assessment of CHD severity. These findings provide a foundation for developing improved diagnostic strategies and may inform personalized therapeutic decisions in the future.

## 2 Materials and methods

### 2.1 Study design and subjects

**Figure 1:**
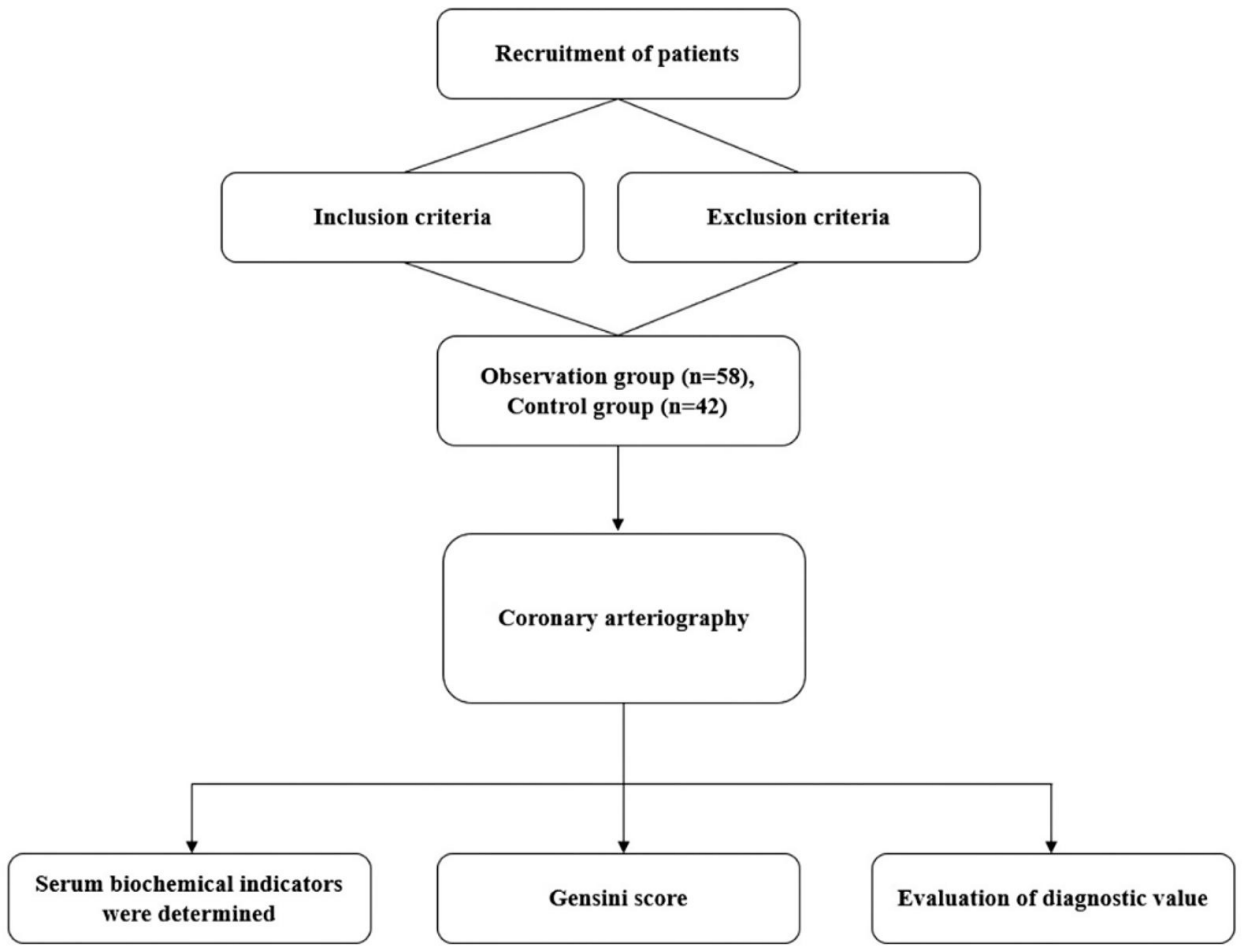
Research flow chart.

During January 2023 and January 2024, 100 patients with symptoms suggestive of CHD, including angina or chest pain, were screened. Patients with decompensated heart failure (NYHA class III–IV) were excluded to reduce potential confounding effects of advanced heart failure on biomarker levels. Eligible participants were assigned to the observation group (OG, *n* = 58) or control group (CG, *n* = 42) according to coronary angiography findings (Figure 1).

The following were the inclusion criteria: (1) be between the ages of 40 and 80; (2) have symptoms suggestive of myocardial infarction or angina pectoris; (3) be in the New York Heart Association (NYHA) functional classes I to II, excluding patients with decompensated heart failure (NYHA III–IV); (4) have not had a major surgery or stroke within the previous six months; (5) have not previously used folic acid antagonists, vitamin B supplements, UA -lowering medications, or glucocorticoids; (6) have not been using antiplatelet drugs (e.g., aspirin, clopidogrel) or anticoagulants (e.g., sodium heparin) within the last two weeks prior to enrollment; (7) duly signed informed permission to be treated.

The following are the exclusion requirements: (1) NYHA functional class III or IV; (2) significant side effects such as right heart failure, hyperthyroidism, heart valve disease, cardiac arrhythmias, and chronic obstructive pulmonary disease; (3) severe disease affecting a major organ (liver, kidneys, or brain); (4) autoimmune disorders, blood disorders, or neoplasms; (5) individuals allergic to the drugs being studied; and (6) a history of cardiac surgery, including but not limited to cardiac stenting or coronary artery bypass grafting; and (7) a history of cerebrovascular disease (e.g., stroke, transient ischemic attack) or lower extremity arterial disease to minimize confounding effects.

Patients with a coronary stenosis burden of at least 50% were defined as having CHD through clinical evaluation, laboratory tests, and CAG (12). Participants were categorized into two cohorts according to the test outcomes: patients with confirmed CHD served as the observation group (OG) (*n* = 58), and individuals without CHD served as the control group (CG) (*n* = 42).

This study is a retrospective observational study. To ensure sufficient statistical power, a *post-hoc* power analysis was conducted, confirming that a total sample size of 100 participants provides >80% power to detect significant differences in Hcy, UA, and HDL-C levels between CHD patients and controls at a significance level of 0.05. Although the study design is relatively simple, the inclusion of multivariable regression, receiver operating characteristic (ROC) analysis, and reclassification metrics (NRI, IDI, *Δ*AUC) enhances the robustness and reliability of our findings. This approach ensures that the observed associations are statistically meaningful and clinically relevant.

For this retrospective study, the data analysis was performed by two experienced radiologists who were blinded to patient identifiers, clinical information, laboratory results, and group assignments. This blinding was ensured by using de-identified datasets, and the radiologists strictly followed a predefined protocol to evaluate coronary angiography and calculate Gensini scores, thereby minimizing potential bias. This study protocol strictly adhered to the Declaration of Helsinki and relevant national ethical standards. The current study was approved by the Ethics Committee of Tianjin Chest Hospital. Written informed consents from all patients were obtained in any experimental work with humans.

### 2.2 Observation indicators

Before collecting blood samples, comprehensive medical histories were obtained from all participants, with particular attention to cardiovascular medications. We recorded all current medications, including statins, angiotensin-converting enzyme inhibitors (ACEIs), angiotensin receptor blockers (ARBs), beta-blockers, calcium channel blockers, and other drugs that might influence lipid metabolism or cardiovascular function. The type, dosage, and duration of statin therapy were specifically documented, noting whether patients were receiving low-, moderate-, or high-intensity statin regimens (defined according to the American College of Cardiology/American Heart Association guidelines).

In our statistical analysis, statin use was included as a covariate in all multivariable regression models examining the relationships between biomarkers and CHD severity. We also performed a sensitivity analysis restricted to statin-naïve patients to confirm the robustness of the associations between Hcy, UA, and HDL-C and Gensini scores independent of statin therapy. We additionally performed subgroup analyses stratified by statin use and conducted sensitivity analyses to assess the robustness of our findings when controlling for medication effects.

To account for the potential confounding effects of diabetes and fasting blood glucose on the associations between Hcy, UA, HDL-C, and CHD severity, these variables were included as covariates in the multivariable linear regression models. Additionally, sensitivity analyses were performed using propensity score matching (PSM) to balance baseline characteristics, including diabetes prevalence and fasting glucose levels, between CHD patients and controls. This approach allowed for a more rigorous evaluation of the independent associations of the biomarkers with CHD severity. To ensure the robustness of our findings, we conducted multiple sensitivity analyses. Multivariable regression models were adjusted for age, gender, BMI, smoking status, statin use, diabetes, and fasting blood glucose to account for potential confounders. Additionally, propensity score matching (PSM) was applied to balance baseline characteristics between CHD patients and controls, including diabetes prevalence, fasting glucose levels, and statin use. Finally, BNP (or NT-proBNP) was included in sensitivity analyses to assess whether its inclusion affects the predictive value of Hcy, UA, and HDL-C. These procedures confirmed the stability of the associations and provided a rigorous evaluation of the independent relationships of the biomarkers with CHD severity.

### 2.3 Serum biochemical indexes

Participants were instructed to fast from 22:00 on the day before the test. After that, 3–5 mL of venous blood samples were drawn from the antecubital vein. Following a 30-minute coagulation period at room temperature, for ten minutes, the samples were centrifuged at 3000 rpm. The processed blood samples were expeditiously sent to the laboratory for forensic examination. HDL-C levels were carried out using immunoturbidimetry, UA levels were assessed by enzymatic assay, and Hcy levels were determined via chemiluminescence. China's Shengzhiyuan Reagents provided the enzyme test kits used to quantify the levels of UA and HDL-C. The quantity of Hcy was determined using a Hcy detector from ADVIA CentaurXP, Siemens Medical Diagnostics, USA. Every reagent was within its designated validity period, and all internal quality control metrics fell below admissible bounds. The analyses adhered to established protocols, and data were documented for *post-hoc* statistical assessment.

### 2.4 Coronary angiography

All participants underwent CAG using a digital x-ray machine (Siemens Axiom Artis Zee Floor). Analysis of the data was conducted by two seasoned radiologists who were unaware of the patient information and other test results.

### 2.5 Coronary artery disease (CAD) score (Gensini score)

To assess CHD severity and quantify coronary stenosis burden, the Gensini scoring system was applied to the CAG results (13, 14). The Gensini score was calculated for all participants (*n* = 100), not only those diagnosed with CHD, enabling a comprehensive assessment of coronary artery stenosis across varying degrees of severity in the study population. The Gensini scoring system is detailed in Table 1.

**Table 1:** Gensini scoring system.

Lesion Characteristics	Scores
Narrowing Percentage	
<25%	1
25% - <50%	2
50% - < 75%	4
75% - < 90%	8
90% - < 99%	16
100% (Complete Occlusion)	32
Lesion Location Score	
Left Main	5
Left Anterior Descending (LAD) - Proximal	2.5
Left Anterior Descending (LAD) - Middle	1.5
Left Anterior Descending (LAD) - Distal	1.0
Left Circumflex (LCx) - Middle/ Distal	1.0
Right Coronary (RCA)	1.0
Small Branches	0.5

The “Narrowing Percentage” column indicates the severity of the coronary artery narrowing, while the “Lesion Location Score” column represents the score for different segments of the coronary arteries. The total Gensini score for a patient is calculated by multiplying the Narrowing Percentage score by the Lesion Location score for each individual lesion.

### 2.6 Statistical analysis

In our statistical analyses, we endeavored to apply rigorous methodologies that clearly associate observed biochemical variations with the Gensini score, representing CHD severity, across all subjects. Each biomarker's influence was assessed using a comprehensive model that considered potential confounders such as age, BMI, and pre-existing conditions, thereby strengthening the validity of our findings. SPSS version 22.0 was the statistical program used for data analysis. Measurement data were provided as the mean adjusted for standard deviation, and the independent sample t-test was used for intergroup comparisons. The count data was analyzed using the chi-square test. A Spearman correlation analysis was performed to assess the relationship between coronary stenosis burden (Gensini scores) and blood chemistry indicators. Using multiple linear regression analysis, components that had a p-value less than 0.2 in the Spearman correlation analysis were chosen for further assessment in order to determine if they are independently associated with the Gensini score. Receiver Operating Characteristic (ROC) curve analysis was used to assess the diagnostic usefulness of blood HDL-C, Hcy, and UA for CHD severity. For statistical significance, a p-value of less than 0.05 was used.

## 3 Results

### 3.1 Baseline characteristics and clinical data of the two groups of subjects

A total of 100 participants were included in this study, with 58 in the OG and 42 in the CG. No appreciable variations were found between the groups in terms of age, sex ratio, body mass index (BMI), smoking status, prevalence of hypertension, proportion of type II diabetes, triglycerides (TG), total cholesterol (TC), left ventricular ejection fraction (LVEF), estimated glomerular filtration rate (eGFR) and creatinine (Scr), indicating that the baseline characteristics of the two groups were comparable and suitable for subsequent comparative analyses. However, significant differences were observed in biochemical indicators related to cardiovascular disease. The OG had higher levels of N-terminal probrain natriuretic peptide (NT-ProBNP), fasting blood glucose, Hcy, and UA compared to the CG (*P* < 0.001). HDL-C levels were considerably lower in the OG (*P* < 0.001). In contrast, LDL-C levels revealed no discernible change between the groups (*P* = 0.330), which may suggest an elevated cardiovascular risk in the OG. Among medication use, 43.00% of participants were receiving statin therapy at enrollment: 55.17% in the OG vs. 26.19% in the CG (*p* = 0.003). None of the participants were statin-naïve. Most were on moderate-intensity therapy (atorvastatin 10–20 mg/day or equivalent). Although baseline characteristics between the observation group (OG) and control group (CG) were largely similar, adding clarity to reported discrepancies is crucial. Notable, even if marginally, was the variation in left ventricular ejection fraction (LVEF) close to the threshold of statistical significance (*p* = 0.051), indicating a need to closely observe cardiac function between groups. Additionally, the significantly different levels of fasting blood glucose between the two groups indicated metabolic discrepancies that could impact cardiac health. Notably, adjustments were made in the analysis to account for potential confounders, including variations in medications received by the participants. Table 2 and Supplementary Table S2 presents the findings.

**Table 2:** Baseline characteristics of participants in the OG and CG.

Variables	Observation Group (*n* = 58)	Control Group (*n* = 42)	*χ*^2^/t	p-value
Age, yr	61 ± 9.8	62.5 ± 9.7	−1.34	0.180
Age subgroup				
40–60 years	20 (34%)	18 (43%)	0.88	0.35
61–80 years	38 (66%)	24 (57%)	0.64	0.43
Male, n(%)	30 (51.72)	23 (54.76)	0.08	0.778
Female, n(%)	28 (48.28)	19 (45.24)	0.06	0.486
BMI, kg/m^^2^	24.3 ± 1.6	23.8 ± 1.5	1.61	0.109
Smoking, n(%)	22 (37.93)	18 (42.86)	0.22	0.639
Non-smoking, n(%)	36 (62.07)	24 (57.14)	0.22	0.639
Hypertension, n(%)	34 (58.62)	25 (59.52)	0.01	0.916
Type II diabetes, n(%)	12 (20.69)	9 (21.43)	0.01	0.931
Previous heart intervention*	0 (0%)	0 (0%)	-	-
LVEF, %	43 ± 6.8	46 ± 7.7	−1.96	0.051
NT-ProBNP, pg/mL	3426.85 ± 1962.46	2125.63 ± 1366.86	7.5	<0.001
Atrial fibrillation, n(%)	10 (17.24)	7 (16.67)	0.003	0.955
Hyperlipidemia, n(%)	20 (34.48)	15 (35.71)	0.007	0.932
Statin use, n(%)	32 (55.17)	11 (26.19)	8.78	0.003
TC, mmol/L	5.00 ± 0.50	4.95 ± 0.45	0.57	0.568
TG, mmol/L	1.72 ± 0.40	1.62 ± 0.38	1.29	0.198
Fasting plasma glucose, mmol/L	7.10 ± 1.20	6.00 ± 1.00	5.25	<0.001
Scr, μmol/L	98.5 ± 14.0	97.0 ± 13.5	0.53	0.596
eGFR, mL/min/1.73m^^2^	66 ± 13.9	66.5 ± 12.8	−0.19	0.849
LDL-C, mmol/L	4.54 ± 0.98	4.38 ± 0.81	0.98	0.330
HDL-C, mmol/L	1.02 ± 0.22	2.05 ± 0.21	−19.23	<0.001
Hcy (*μ*mol/L)	18.34 ± 7.52	12.42 ± 6.78	4.58	<0.001
UA (μmol/L)	446.89 ± 95.42	385.89 ± 72.41	3.26	0.001

Except for LVEF and fasting plasma glucose, no discernible variations existed in the baseline data of the patients in the two groups (*p* < 0.05).

In the control group (non-CHD participants), all individuals were confirmed to have less than 50% coronary artery stenosis based on coronary angiography, indicating the absence of clinically significant stenosis. Minor or subclinical narrowing was observed in some participants, which resulted in Gensini scores slightly above zero. The descriptive statistics for the control group are as follows: mean ± SD = 2.3 ± 3.1, range = 0–10. These results demonstrate that, while all controls were free from clinically meaningful stenosis, minor variations in coronary artery structure were appropriately captured by the Gensini scoring method.

### 3.2 Correlation between gensini score and variables

To further delineate the relationship between CHD severity and clinical characteristics, Spearman rank correlation analysis was performed for all enrolled participants (Table 3). Several variables showed significant associations with the Gensini score. Renal function markers demonstrated the strongest correlations, with serum creatinine (Scr) exhibiting a moderate positive correlation (r = 0.411, *p* < 0.001), while eGFR displayed an inverse association (r = −0.318, *p* < 0.001). Metabolic and cardiac biomarkers, including homocysteine (Hcy), uric acid (UA), NT-proBNP, and fasting plasma glucose were also positively correlated with the Gensini score (r = 0.252–0.314, all *p* < 0.01). In addition, age and BMI showed weak-to-moderate positive correlations. Notably, HDL-C exhibited a significant negative correlation (r = –0.324, *p* < 0.001), underscoring its potential protective role against the progression of coronary atherosclerosis.

**Table 3:** Relationship between gensini score and Variable.

Variable	Spearman Correlation Analysis	
	r	P
Serum Creatinine (Scr)	0.411	<0.001
eGFR	−0.318	<0.001
Hcy	0.314	<0.001
UA	0.307	<0.001
N-terminal pro B-type Natriuretic Peptide (NT-ProBNP)	0.294	<0.001
Fasting Plasma Glucose	0.252	0.001
Type II Diabetes	0.248	0.001
BMI	0.231	0.003
Age	0.227	0.003
Age subgroup 40–60 years	0.192	0.045
Age subgroup 61–80 years	0.238	0.021
Atrial Fibrillation	0.153	0.049
Current statin use	−0.165	0.040
Hypertension	0.148	0.045
LVEF	0.127	0.104
Low-Density Lipoprotein Cholesterol (LDL-C)	0.100	0.202
Smoking	0.095	0.225
HDL-C	−0.324	<0.001
Hyperlipidemia	0.087	0.267
TG	0.031	0.694
TC	0.001	0.994
Gender	−0.009	0.910

This table demonstrates the relationship between various factors and the Gensini score, which is used to assess the severity of CAS. Variables are ordered according to their correlation with the Gensini score. A positive correlation (r) suggests that higher levels of the variable are associated with more severe stenosis, while a negative correlation suggests the opposite.

Multivariable linear regression analysis (Table 4) further evaluated the independent contribution of these variables. Model 1, which included core clinical predictors, explained 31% of the variance in the Gensini score (R^2^ = 0.31, *p* < 0.001). After incorporating Hcy, UA, and HDL-C (Model 2), the explanatory power increased to 48% (R^2^ = 0.48, *p* < 0.001), indicating a modest incremental value from these biochemical markers. The increase in explanatory power from Model 1 (clinical predictors, R^2^ = 0.31) to Model 2 (clinical predictors + biomarkers, R^2^ = 0.48) was evaluated using an *F*-test for *Δ*R^2^. The result (F = 9.92, *P* < 0.001) indicates that the addition of Hcy, UA, and HDL-C significantly improves the model's ability to explain the variance in Gensini scores beyond traditional clinical predictors.

**Table 4:** Multistep linear regression models identifying independent predictors of gensini score.

Variable	Model 1 – Clinical predictors only	Model 2 – Clinical predictors + biomarkers
Age	*β* = 0.178, *p* = 0.029	*β* = 0.142, *p* = 0.041
Type II diabetes	β = 0.154, *p* = 0.034	β = 0.112, *p* = 0.072
Age subgroup 40–60 years	β = 0.125, *p* = 0.058	β = 0.110, *p* = 0.067
Age subgroup 61–80 years	β = 0.186, *p* = 0.021	β = 0.148, *p* = 0.034
eGFR	β = −0.182, *p* = 0.024	β = −0.145, *p* = 0.048
NT-proBNP	β = 0.180, *p* = 0.011	β = 0.152, *p* = 0.020
UA	—	β = 0.149, *p* = 0.044
Hcy	—	β = 0.278, *p* = 0.017
HDL-C	—	β = −0.275, *p* = 0.016
Gender (Male/Female)	—	β = 0.118, *p* = 0.072
Smoking (Yes/No)	—	β = 0.095, *p* = 0.085
Model R^2^	0.31	0.48
Model p-value	<0.001	<0.001

Model 1 served as the baseline clinical model and incorporated core predictors of coronary artery disease severity—age, type II diabetes, eGFR, and NT-proBNP—selected *a priori* based on their well-established clinical relevance. To assess the added predictive contribution of the biochemical biomarkers under investigation, Model 2 was constructed by incorporating UA, Hcy, and HDL-C into Model 1. Multivariable linear regression yielded β coefficients and p-values for each predictor, with positive coefficients indicating greater stenosis severity and negative coefficients indicating an inverse association. Model R^2^ values quantify the proportion of explained variation in the Gensini score. Model performance was evaluated using *F*-tests. The improvement observed after adding UA, Hcy, and HDL-C demonstrates meaningful incremental predictive value, consistent with the reclassification and discrimination statistics (NRI, IDI, and *Δ*AUC) presented in Table 6.

In this adjusted model, Hcy and UA remained positively associated with coronary stenosis burden, whereas HDL-C demonstrated an inverse association, reinforcing their biological relevance in the pathogenesis and progression of coronary artery disease.

Sensitivity analyses confirmed the robustness of our findings. After adjustment for age, gender, BMI, smoking status, statin use, diabetes, and fasting blood glucose, Hcy, UA, and HDL-C remained independently associated with Gensini scores (all *P* < 0.05). Propensity score matching, balancing key baseline characteristics between CHD patients and controls, yielded consistent results. Moreover, the inclusion of BNP (or NT-proBNP) as a covariate did not materially change the associations, indicating that the primary findings are stable and not substantially influenced by additional cardiovascular biomarkers. Similarly, the sensitivity analysis restricted to statin-naïve patients confirmed that the associations between these biomarkers and coronary stenosis burden remained consistent, indicating that the primary findings are robust and not substantially influenced by statin therapy.

To validate the robustness of variable selection, best subset regression was performed, with results presented in Supplementary Figure S1. The variable inclusion plot (VIP) revealed that Hcy, UA, and HDL-C consistently achieved the highest adjusted R^2^ among all candidate predictors, highlighting their strong model contributions. The Bayesian Information Criterion (BIC) curve further demonstrated that the three-predictor model achieved the lowest BIC value, representing an optimal balance between model fit and parsimony. These findings align well with the multivariable regression results and collectively support the central role of Hcy, UA, and HDL-C in predicting coronary stenosis burden.

Overall, the Gensini score was not only associated with traditional cardiovascular risk factors and cardiac-renal functional indices but also strongly linked to metabolic biomarkers such as Hcy, UA, and HDL-C. Their combined assessment substantially improved the explanatory capacity for CHD severity and may enhance clinical risk stratification.

### 3.3 Stratified analysis based on statin use

Statin users (*n* = 35) had significantly lower Hcy levels (11.2 ± 3.4 μmol/L) compared with non-users (*n* = 23, 14.8 ± 4.1 μmol/L, *p* = 0.002). UA levels were also lower in statin users (320 ± 65 μmol/L) vs. non-users (365 ± 70 μmol/L, *p* = 0.01). HDL-C was slightly higher in statin users (1.25 ± 0.35 mmol/L) compared with non-users (1.15 ± 0.30 mmol/L, *p* = 0.18), but this difference was not statistically significant. Gensini scores were lower in statin users (28.5 ± 12.7) than in non-users (35.2 ± 14.1, *p* = 0.03), suggesting a potential protective effect of statins. In multivariable regression, statin therapy remained an independent predictor of lower Gensini score after adjusting for age, sex, BMI, hypertension, and diabetes (*β* = –0.22, *p* = 0.04). These results provide specific evidence of the effect of statins on biomarkers and CHD severity.

### 3.4 Clinical significance of combined detection of Hcy, HDL-C, and UA in the diagnosis of CHD

**Figure 2:**
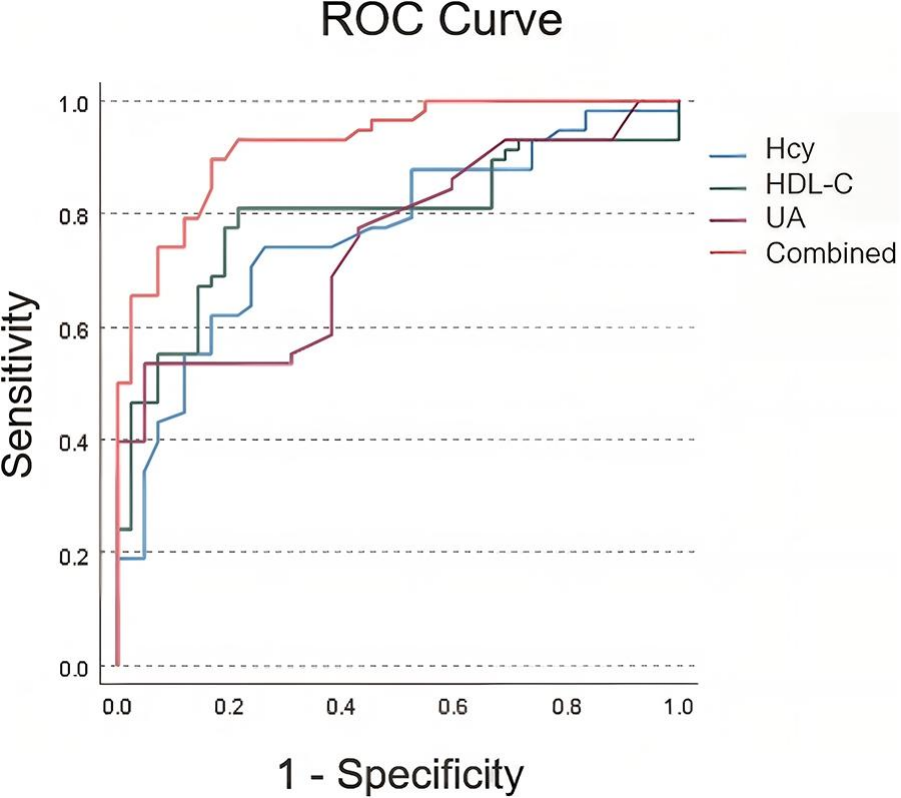
Receiver operating characteristic (ROC) curves for Hcy, HDL-C, UA, and their combination in diagnosing coronary artery stenosis.

To evaluate the diagnostic value of the metabolic biomarkers under investigation, the performance of Hcy, HDL-C, UA, and their combined detection model was assessed using ROC analysis (Table 5; Figure 2). Individually, Hcy demonstrated the highest diagnostic accuracy among the single biomarkers (AUC = 0.794, 95% CI: 0.706–0.882), followed by HDL-C (AUC = 0.767) and UA (AUC = 0.749). All three biomarkers showed statistically significant discriminatory ability forCHD severity (*p* < 0.001). Optimal cutoff values derived from Youden's index yielded sensitivities ranging from 59.7% to 69.5% and specificities from 68.2% to 72.4%, indicating moderate standalone diagnostic capability.

**Table 5:** Diagnostic performance of Hcy, HDL-C, UA and their combination for the diagnosis of CHD.

Biomarkers	AUC	p-value	95% CI	Cut-off Value	Sensitivity (%)	Specificity (%)	Youden Index
Hcy (μmol/L)	0.794	<0.001	0.706–0.882	15.47 μmol/L	69.5	72.4	0.419
HDL-C (mmol/L)	0.767	<0.001	0.677–0.857	1.42 mmol/L	64.3	70.6	0.349
UA (μmol/L)	0.749	<0.001	0.659–0.839	412.68μmol/L	59.7	68.2	0.279
Hcy + HDL-C + UA	0.803	<0.001	0.756–0.846	—	69.8	74.2	0.440

When Hcy, HDL-C, and UA were incorporated into a combined detection model, the AUC increased to 0.803, representing a modest improvement in performance compared with individual biomarkers. The combined model achieved a sensitivity of 69.8% and a specificity of 74.2%, along with the highest Youden index (0.440), suggesting improved classification efficiency for identifying individuals with coronary stenosis burden.

Beyond ROC discrimination, reclassification analyses further demonstrated the clinical benefit of the combined biomarker strategy. As shown in Table 6, the integrated model yielded significant improvements in both NRI (overall NRI = 0.24, *p* = 0.001) and IDI (IDI = 0.065, *p* = 0.002), indicating enhanced risk stratification for both CHD cases and controls. The *Δ*AUC of 0.084 (*p* = 0.004) confirms the modest incremental discriminatory value conferred by adding Hcy, HDL-C, and UA to the baseline clinical model. Supplementary Table S1 further illustrates the movement of subjects across risk categories, revealing a net improvement of 15.5% among events and 7.2% among non-events.

**Table 6:** Net reclassification improvement (NRI), integrated discrimination improvement (IDI), and *Δ*AUC.

Metric	Estimate	95% CI	p-value
NRI (Overall)	0.24	0.10–0.38	0.001
NRI for events	0.16	0.05–0.28	0.004
NRI for non-events	0.08	0.01–0.17	0.028
IDI	0.065	0.030–0.110	0.002
*Δ*AUC	0.084	0.032–0.129	0.004

**Figure 3:**
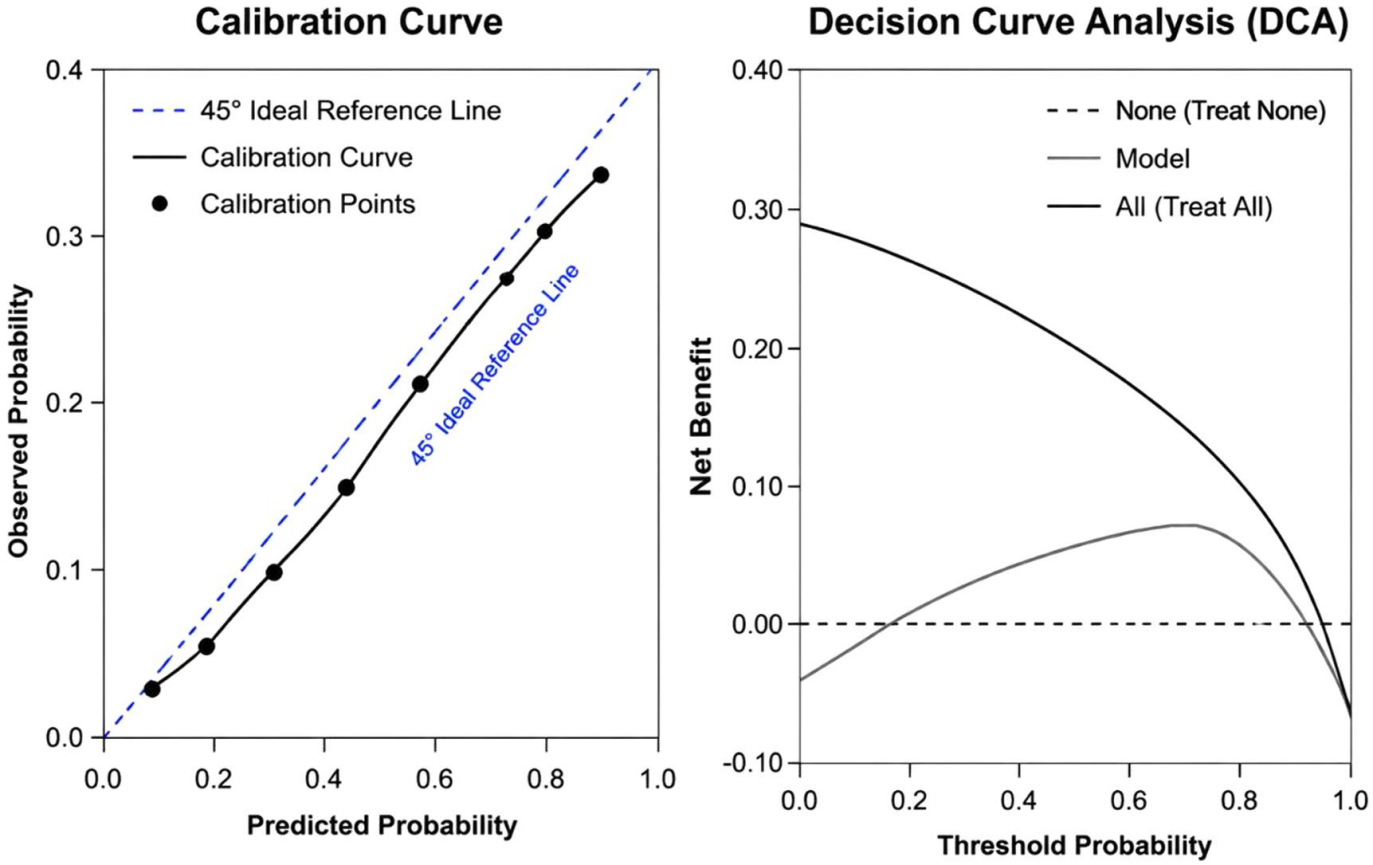
Calibration and decision curve analysis of the predictive model based on HDL-C, Hcy, and UA. **(A)** Calibration curve assessing the agreement between predicted and observed probabilities of coronary artery disease (CAD). The solid line represents the model's calibration performance after bootstrap resampling (1,000 repetitions), the dots indicate the observed risk within each decile, and the dashed line denotes the ideal reference line (perfect calibration at 45°). **(B)** Decision curve analysis (DCA) illustrating the net clinical benefit of the predictive model compared with the “None” and “All” strategies across a range of threshold probabilities. The model demonstrates a consistently higher net benefit, supporting its potential clinical utility for risk stratification.

Model calibration and decision curve analysis supported the robustness and clinical utility of the combined biomarker approach (Figure 3). The calibration plot demonstrated good agreement between predicted and observed risk, while the decision curve showed that the combined model provided greater net clinical benefit across a broad range of threshold probabilities compared with the “treat-all” and “treat-none” strategies. Collectively, these findings highlight that simultaneous assessment of Hcy, CysC, and UA offers modest but meaningful diagnostic and prognostic value, outperforming single-marker approaches and contributing substantially to individualized risk stratification in CHD severity.

## 4 Discussion

This study's goal was to investigate the connection between UA, HDL-C, and Hcy and CHD severity and CAS, and to evaluate their value as non-invasive diagnostic tools. According to our findings, the simultaneous measurement of serum HDL-C, Hcy, and UA levels has a high sensitivity and specificity for the diagnosis of CHD and may be used as a useful biomarker for assessing coronary stenosis burden (15).

The relationship between Hcy and CHD has been widely studied, and the Hcy level in the OG in this study was significantly higher than that in the CG, which is consistent with previous studies (16). Because Hcy promotes atherosclerosis formation and damages endothelial function through oxidative stress mechanism, thereby increasing the risk of CAD. HDL-C is commonly referred to as “good” cholesterol because it helps prevent atherosclerosis. Consequently, lower HDL-C levels are often connected to a heightened risk of CAD (17, 18). Our investigation revealed that HDL-C levels were considerably lower in the OG, reinforcing the link between reduced HDL-C levels and increased CHD risk (4). UA acts as an endogenous antioxidant, but elevated levels of UA are associated with a higher risk of CAD (19). This association may stem from the relationship between high UA levels and impaired endothelial function, as well as increased inflammatory status. Consistent with this, our results demonstrated considerably higher UA levels in the OG.

Notably, our analysis revealed a considerable positive correlation between serum HDL-C, Hcy, and UA levels and the Gensini score. This indicates that these biochemical markers can be utilized not only for diagnosing CHD but also for assessing CHD severity. Multiple linear regression analysis further confirmed that Hcy, UA, and HDL-C significantly impact the Gensini score as independent variables. These findings highlight the value of combined measurement of Hcy, UA, and HDL-C in clinical practice, as they provide critical insights into coronary stenosis burden, thereby aiding clinical decision-making. Additionally, ROC curve analysis demonstrated that the combined detection of UA, Hcy and HDL-C yields a higher AUC for diagnosing CHD compared to individual markers. This suggests that the combined use of these biomarkers can significantly enhance the accuracy of CHD diagnosis. While CAG remains the gold standard for diagnosing CHD, its invasiveness and cost underscore the utility of combining Hcy, HDL-C, and UA as a simple, effective, and non-invasive alternative. Although these biomarkers are not substitutes for coronary angiography (CAG), their combined measurement may serve as an adjunctive indicator of CHD severity in situations where CAG is delayed or unavailable.

It is important to note that the R^2^ values in our multivariable models (0.31–0.48) and the *Δ*AUC improvement (0.084) indicate that additional factors influencing CHD severity were not captured in the current analysis. Coronary artery disease is a multifactorial condition, and while Hcy, UA, and HDL-C were independently associated with Gensini scores, other important variables, such as genetic predisposition, inflammatory markers, lifestyle factors, and additional metabolic parameters were not included. Despite the modest R^2^ and *Δ*AUC, the combined assessment of these three biomarkers still provides statistically significant improvement over single-marker models and demonstrates practical clinical utility as non-invasive indicators for CHD severity. Future studies incorporating a broader range of predictors are warranted to enhance the explanatory power and diagnostic accuracy of the models. Although BNP was independently associated with Gensini scores, the primary focus of this study on Hcy, UA, and HDL-C remains valid. The inclusion of BNP in sensitivity analyses did not change the predictive value of these biomarkers, supporting their utility as non-invasive indicators of CHD severity.

Notably, we observed a modest negative correlation between current statin use and Gensini score (r = −0.165, *p* = 0.040), indicating that patients receiving statin therapy tended to have slightly lower CHD severity. This highlights the potential confounding effect of statins, which are known to reduce Hcy and UA levels and modestly increase HDL-C. To account for this, statin use was included as a covariate in all multivariable regression models. Additionally, a sensitivity analysis restricted to statin-naïve patients confirmed that the associations between Hcy, UA, and HDL-C and Gensini scores remained consistent, demonstrating that the primary findings are robust and not substantially influenced by statin therapy.

This study has several limitations that merit consideration:

1. Single-center, small-sample design: The study was conducted at a single center (Tianjin Chest Hospital) with a total of 100 patients (58 cases in the coronary heart disease group and 42 controls), which may limit the representativeness and generalizability of the findings across broader populations. This single-center cohort may not fully reflect population characteristics across different regions, ethnicities, genetic backgrounds, or healthcare environments. The relatively small sample size may reduce statistical power, potentially affect the stability and reliability of regression models and ROC analyses, and increase the influence of random errors. A *post-hoc* power analysis confirmed that this sample provides >80% power to detect significant differences in Hcy, UA, and HDL-C levels, but larger cohorts are needed to strengthen the robustness of the conclusions.
2. Cross-sectional study design: The cross-sectional design limits the interpretability of the observed associations, which cannot be extrapolated to prognostic implications or predictive utility for subsequent cardiovascular events. Because only baseline data were collected, this design cannot establish causality or determine the temporal sequence of biomarker changes and disease onset or progression (e.g., whether elevated Hcy precedes CHD or results from disease progression). It also does not allow evaluation of dynamic biomarker changes over time or their predictive value for future cardiovascular events, limiting the clinical guidance that can be drawn from the results.
3. Single baseline biomarker measurements: Biomarkers of interest Hcy, HDL-C, and UA were measured only once at baseline without dynamic monitoring, which precludes the assessment of intra-individual variability, temporal changes, or post-treatment responses. A single measurement may also be influenced by short-term factors, such as pre-test diet, emotional state, or transient health conditions, making it difficult to accurately reflect the patient's long-term stable status and potentially affecting the reliability of biomarker-CHD severity associations. While statistical methods including multivariable regression, ROC analysis, and reclassification metrics (NRI, IDI, *Δ*AUC) were applied to enhance reliability, the limited sample size and single measurements still pose constraints on the robustness of findings. Therefore, the study cannot reflect fluctuation patterns within individuals, responses to interventions such as statins or urate-lowering agents, or associations between biomarker changes and progression/reversal of coronary stenosis burden. Consequently, temporal trajectories could not be characterized, and causal relationships cannot be inferred from the present findings. Additionally, the results of subgroup analyses stratified by age, gender, CHD severity, or statin use were not fully conducted or detailed, limiting the ability to assess the modulatory role of these factors on biomarker-disease associations and the applicability of conclusions to specific populations.
4. Limitations of coronary artery stenosis assessment: The severity of CHD was quantified using the Gensini score, a widely adopted angiographic metric that evaluates only the degree and location of coronary stenosis burden. This scoring system does not incorporate key pathological features such as plaque morphology (e.g., eccentric or concentric plaques), plaque composition (e.g., lipid core, degree of calcification), or plaque stability (e.g., vulnerable plaques), which modern cardiovascular research has identified as core predictors of acute cardiovascular events. As a result, the use of the Gensini score limits the ability to establish associations between biomarkers and these critical pathological features, reducing the precision of clinical risk stratification. Integrating intravascular imaging modalities such as IVUS or OCT would allow for a more nuanced characterization of coronary pathology and could refine the correlations observed in this study.
5. Potential uncontrolled confounding factors: Although adjustments were made for some confounding factors, such as statin use, age, and diabetes, other potential confounders including dietary structure, exercise habits, folate/B-complex vitamin intake, and genetic polymorphisms were not fully assessed. These unmeasured factors could simultaneously influence biomarker levels and CHD severity, resulting in residual confounding bias in the association analysis. Additionally, the timing of blood sample processing (e.g., interval from centrifugation to testing) was not fully documented, and the stability of Hcy and UA during storage was not independently validated, which may introduce detection bias. This represents an additional methodological limitation.

Finally, validation in larger, multiethnic, multicenter cohorts is necessary to confirm the broader applicability and robustness of these findings. Future studies should focus on more comprehensive subgroup analyses, particularly by age, gender, and CHD severity, to provide targeted guidance for specific populations.

In conclusion, by adopting rigorous statistical analysis and clear, concise language throughout our study, we have highlighted significant correlations between uric acid, HDL-cholesterol, homocysteine levels, and CHD severity as gauged by the Gensini score. Our research supports the utility of these biomarkers in assessing coronary stenosis burden and potentially guiding clinical interventions. Future studies should adopt longitudinal designs with repeated biomarker measurements to clarify causality, evaluate dynamic changes over time, and assess the predictive value of these biomarkers for disease progression and prognosis in larger, more diverse populations.

## Data Availability

The raw data supporting the conclusions of this article will be made available by the authors, without undue reservation.
