# Supplementary material for: Association of uric acid, HDL-C, and homocysteine with coronary artery disease and stenosis
Source: Front Cardiovasc Med. 2026 Jul 20;13:1765538. doi: 10.3389/fcvm.2026.1765538 (PMC13429707; doi:10.3389/fcvm.2026.1765538)
Supplement: Supplementary file 1 [file Datasheet1.docx]

**Supplementary tables and figure**

**Supplementary Table 1** Reclassification Movement After Adding UA, Hcy, and HDL-C to the Base Model

| Group | No. of patients | Moved to Higher Risk | Moved to Lower Risk | Net Improvement |
| --- | --- | --- | --- | --- |
| Events (CAD patients, n = 58) | 58 | 18 (31.0%) | 9 (15.5%) | +9 (15.5%) |
| Non-events (Controls, n = 42) | 42 | 4 (9.5%) | 7 (16.7%) | +3 (7.2%) |
| Overall NRI | — | — | — | 0.24 |

**Supplementary Table 2**. Cardiovascular Medication Use and Typical Dose/Intensity (Demonstration Only)

| Medication category | OG (n=58) | CG (n=42) | Statistic | p-value | Dose / intensity (typical range) |
| --- | --- | --- | --- | --- | --- |
| Statins (any) | 32 (55.17%) | 11 (26.19%) | χ² = 8.78 | 0.003 | *Atorvastatin 10–20 mg/day; Rosuvastatin 5–10 mg/day* |
| ACE inhibitors | 18 (31.0%) | 10 (23.8%) | χ² = 0.68 | 0.410 | *Enalapril 5–10 mg/day* |
| ARBs | 20 (34.5%) | 12 (28.6%) | χ² = 0.45 | 0.502 | *Valsartan 80–160 mg/day* |
| β-blockers | 30 (51.7%) | 14 (33.3%) | χ² = 3.77 | 0.052 | *Metoprolol 25–50 mg/day* |
| CCB | 26 (44.8%) | 15 (35.7%) | χ² = 0.95 | 0.330 | *Amlodipine 5–10 mg/day* |
| Diuretics | 12 (20.7%) | 6 (14.3%) | χ² = 0.72 | 0.397 | *HCTZ 12.5–25 mg/day* |
| Antiplatelet therapy | 45 (77.6%) | 18 (42.9%) | χ² = 13.8 | <0.001 | *Aspirin 75–100 mg/day* |
| UA-lowering drugs | 0 | 0 | — | — | *Excluded per protocol* |
| Vitamin B / folate supplements | 0 | 0 | — | — | *Excluded per protocol* |
| Glucocorticoids | 0 | 0 | — | — | *Excluded per protocol* |

**Supplementary Figure S1**


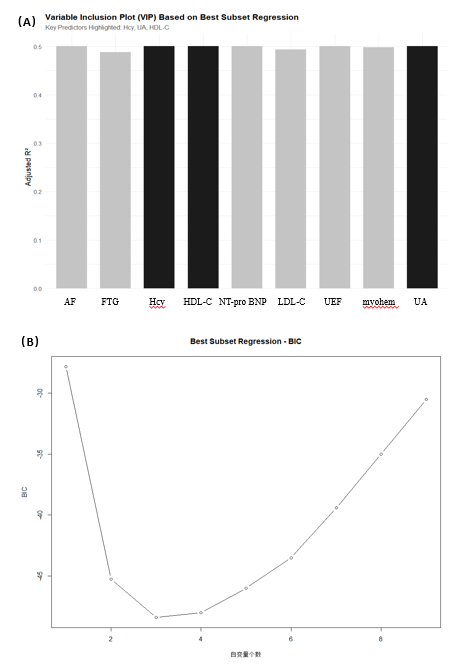


**Supplementary Figure S1. Best subset variable selection via adjusted R² and BIC.** (A) Variable inclusion plot (VIP) based on best subset regression. Bars represent the maximum adjusted R² achieved by models that include each predictor. The key predictors Hcy, UA and HDL-C are highlighted in black, indicating their strong contribution to model performance. (B) Bayesian Information Criterion (BIC) of the best subset models as a function of the number of predictors. The minimum BIC is observed for the three-predictor model, supporting the selection of Hcy, UA and HDL-C in the final regression model.
